# Examining Canadian Trauma Centres’ Analgesic Protocols for Rib Fractures

**DOI:** 10.5811/westjem.24945

**Published:** 2025-09-25

**Authors:** Sammie Yu, Petrease Patton, Kelly Vogt, Fran Priestep, Richard Hilsden, Shane Smith, Ian Ball

**Affiliations:** * Western University, Faculty of Science, London, Ontario, Canada; † Western University, Schulich School of Medicine and Dentistry, London, Ontario, Canada; ‡ Western University, Department of Medicine, London, Ontario, Canada; § Western University, Department of Surgery, London, Ontario, Canada; ¶ Western University, Office of Academic Military Medicine, London, Ontario, Canada; || Victoria Hospital, Trauma Program, London Health Sciences Centre, London, Ontario, Canada; # Western University, Department of Epidemiology and Biostatistics, London, Ontario, Canada

## Abstract

**Introduction:**

Rib fractures are common in patients with blunt thoracic trauma, and their associated pain causes significant morbidity and mortality. Adequate analgesia is crucial to prevent rib fracture-associated pulmonary complications. However, current analgesic modalities have drawbacks, and the optimal analgesia protocol remains elusive. Intravenous (IV) lidocaine infusions have a well-established safety profile and efficacy in other patient populations and may benefit patients with traumatic rib fractures. To better understand current practices and to inform the design of a multi-centre trial, we believe that a study to determine Canadian trauma centres’ current analgesic practices is warranted. This study describes the current familiarity and use of IV lidocaine infusions for management of rib fracture pain. Secondary outcomes included the identification of common Canadian analgesic protocols for rib fractures and willingness to participate in a future multi-centre trial of lidocaine for these traumatic injuries.

**Methods:**

We distributed an online survey to 14 Canadian trauma centres. Study questions were designed to address four themes: trauma centre characteristics; pain management strategies; current use of IV lidocaine infusions; and interest in future study participation. The analysis included a frequencies analysis and a thematic analysis of descriptions.

**Results:**

The medical directors of 12 trauma centres (85%) responded. Six of those centres (50%) experience > 450 annual trauma admissions with Injury Severity Scores > 12. Six sites (50% of respondents) have a rib-fracture analgesic protocol. Four centres (33% of respondents) frequently use IV lidocaine for rib fractures, and 10 (83% of respondents) believe further research with IV lidocaine is needed.

**Conclusion:**

Canadian trauma centres’ current practices for rib-fracture pain management are variable. Prospective work is needed to evaluate IV lidocaine as an analgesic for traumatic rib fractures.

## INTRODUCTION

Traumatic rib fractures are relatively common in all trauma patients, with an incidence of 10%.^1^ The incidence increases to nearly 40% for blunt thoracic trauma patients.^2^ Among those patients, the elderly population suffers a mortality rate of 68%, while the general population experiences a mortality rate of approximately 20%.^2^ Medical comorbidities in elderly patients increase their mortality from rib fractures.^3^,^4^ Overall, rib fractures remain common in all trauma patients of all ages and cause significant morbidity and mortality.

Rib fracture-associated morbidity and mortality is due to the pain that patients experience,^2^,^5^–^7^ particularly with movement and breathing.^8^ Pain from rib fractures causes patients to cough less and clear secretions less often and less effectively. Retained secretions can increase bacterial colonization and, consequently, promote pneumonia.^5^–^7^,^9^ Rib fractures can also cause muscle spasms that cause atelectasis and reduced respiratory function. Patients may experience hypoxemia, increased shunt fraction, and ultimately respiratory failure requiring intubation and mechanical ventilation.^5^–^7^,^9^

Analgesia is essential to prevent life-threatening pulmonary complications and mitigate rib fracture-associated morbidity and mortality.^10^ However, there is currently no optimal recommendation for pain management.^6^ Current analgesic practices include acetaminophen, non-steroidal anti-inflammatory drugs (NSAID), intravenous (IV) opioids, and epidural catheters.^10^ Less common approaches include intercostal nerve blocks, intrapleural or intrathecal anesthesia, and thoracic paravertebral blocks.^11^,^12^

Current protocols for treatment of rib fractures present challenges, including inadequate analgesia provided to patients. The current standard for the London Health Sciences Centre in Ontario, Canada, is IV opioids; however, opioids have a narrow therapeutic index in rib fractures. High doses of opioids will reduce respiratory drive and secretion clearance, thereby exacerbating conditions contributing to mortality.^11^ In contrast, the Eastern Association for the Surgery of Trauma (EAST) guideline on pain management in blunt thoracic trauma conditionally recommends the use of epidural analgesia instead of opioids.^13^ However, common conditions in trauma patients such as fever and altered mental status may prevent epidural analgesia administration.^12^ Moreover, a review of retrospective trials highlights a use rate of epidural analgesia < 20% due to practical challenges such as anesthetist availability and patient cooperation.^14^

The ideal protocol for a safe and effective analgesic modality for rib fracture patients remains elusive. Currently, guidelines for pain management do not include IV lidocaine infusions, despite literature supporting its use in thoracic surgery patients.^15^–^17^ While IV lidocaine has not been extensively studied in trauma patients, a meta-analysis demonstrated its efficacy in reducing pain scores and the length of hospital stays after surgery in other patient populations.^18^ Furthermore, previous investigations showed that IV lidocaine might be the best alternative when epidurals are contraindicated, refused, unavailable or ineffectual. Compared to epidurals, IV lidocaine demonstrates no difference in subsequent pain intensity or postoperative complications.^19^,^20^ Most notably, a single-centre, randomized controlled trial has demonstrated the potential of lidocaine as a beneficial analgesic for patients with rib fracture.^8^ The study used the general IV dosing of 1–4 milligrams per kilogram (mg/kg) as a bolus, followed by 1–4 mg/kg/hour as an infusion.^8^

**Population Health Research Capsule**

What do we already know about this issue?

*Rib fractures cause significant pain and complications. IV lidocaine has a well-established safety profile, but its use for rib fracture pain is not widespread*.

What was the research question?

*What are Canadian trauma centres’ current analgesic practices and attitudes toward IV lidocaine for rib fracture pain?*

What was the major finding of the study?

*33% of trauma centres use IV lidocaine for rib fractures, and 83% support further research on its potential efficacy*.

How does this improve population health?

*This study highlights the potential of IV lidocaine for rib fracture pain. Supporting research could standardize management and reduce pain-induced complications*.

Systemic administration of IV lidocaine offers advantages over local infiltration of lidocaine in addressing broader pain pathways from rib fractures injuries. Rib fractures induce pain that can radiate from the site of fracture to surrounding muscles and dermatomes, exacerbating patient morbidity and mortality, especially during movement.^8^ Systemic lidocaine infusions have been shown to improve pain management by their anti-hyperalgesic properties.^21^,^22^ By contrast, IV lidocaine exerts its pharmacological action through sodium channel blockade in neural tissues. Studies also suggest systemic lidocaine may reduce or prevent the proliferation of sodium channels, thus interrupting neuronal transmission, particularly in traumatized tissues. 21,23 Additionally, studies have shown that IV lidocaine can decrease systemic inflammatory markers, demonstrating significant anti-inflammatory properties.^24^ Given these effects, combined with our understanding of rib fracture-associated mortality related to pain,^2^,^5^–^7^ as well as evidence supporting the effectiveness of IV lidocaine in thoracic surgery,^15^–^17^ we sought to investigate the use of IV lidocaine to blunt pain perception and reduce pain-induced, rib fracture complications as suggested by related studies.^8^,^19^,^20^

We surveyed current the medical directors of Canadian trauma centres regarding their current practices and experiences with rib fracture pain management, including their familiarity with and use of IV lidocaine infusions in Canadian trauma patients. Secondary outcomes of the study included a measure of the most common analgesic protocols for thoracic traumas and of those centres’ willingness to participate in future research.

## METHODS

We developed an online survey for electronic distribution across 14 major Canadian trauma centres using REDCap (Research Electronic Data Capture) software hosted by the Lawson Research Institute. REDCap is an encrypted online application used to collect research data. We developed a 31-item descriptive survey with an expected completion time of < 25 minutes, which was distributed to a convenience sample of the medical directors of 14 major Canadian trauma centres. Each respondent was given a unique, single-use link and was required to enter the name of the trauma centre they were representing to ensure no duplicated responses. There were no additional inclusion or exclusion criteria.

The survey was designed to address four major themes: trauma centre characteristics and demographics; current or preferred pain management protocol strategies; current use of IV lidocaine infusion in trauma patients, including rib fracture patients; and the medical directors’ opinions regarding the importance of IV lidocaine in trauma patient research and interest in participating in a multi-centre randomized controlled trial to examine the effectiveness of IV lidocaine infusions. Study questions were posed as a variety of multiple-choice questions, including Likert scale, rating-scale, ratio-scale and dichotomous responses, and situation-based questions. Situation-based questions included descriptive opportunities to allow elaboration, where appropriate, using free-form text boxes.

Completed response data was recorded using REDCap software. The collected data was only made available to members of the research team. Following data collection, responses were aggregated before analysis. The team collectively performed a narrative synthesis of the frequencies, patterns, variation, and consensus across treatment centres and conducted a thematic analysis of the descriptive answers to determine similarities and differences from the general trend. The study was unblinded to compare inter-provincial practice variation.

The survey was distributed in February 2023 through email to the trauma centre medical directors, or their delegates, of the following academic hospitals located across five Canadian provinces: British Columbia (Royal Columbian Hospital, Vancouver General Hospital, Victoria General Hospital); Alberta (University of Alberta Hospital, Foothills Medical Centre); Saskatchewan (Royal University Hospital, Regina General Hospital); Ontario (Hamilton Health Sciences, London Health Sciences Centre, and The Ottawa Hospital, Kingston Health Sciences Centre, Sunnybrook Health Sciences Centre, St. Michael’s Hospital); and Nova Scotia (Halifax Health). A reminder email was sent three weeks after the initial contact to encourage survey completion.

## RESULTS

### Site Characterization

The survey was completed by 12 of 14 (85%) medical directors of Canadian trauma centres. Survey responses regarding trauma centre characteristics and demographic information are presented in Table 1. When surveyed, six (50%) of the responding centres reported > 450 severe trauma admissions annually (Table 1).

### Pain Management

**Figure 1:**
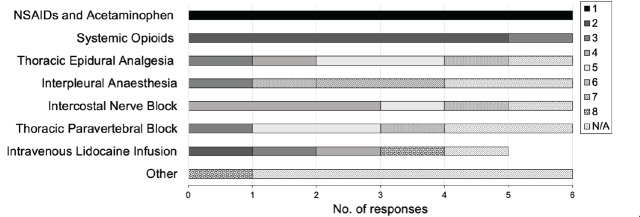
Rib fracture pain management in Canadian trauma centres without a protocol. Data represents the ranking preference of pain management strategies based on surveyed rating scale responses (N = 6; 50%), with 1 indicating the highest preference. *NSAIDs*, non-steroidal anti-inflammatory drugs; *N/A*, treatment strategies that were not used at the trauma centre were not ranked; *Other*, 1 centre reported using ketamine.

**Figure 2:**
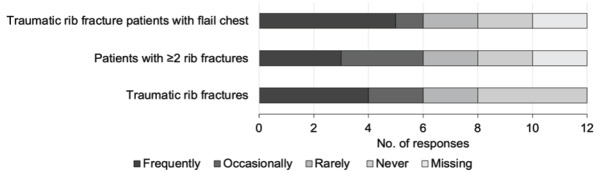
Current use of intravenous (IV) lidocaine infusions in Canadian trauma centres. Data shown represents centres’ Likert scale responses addressing the frequency of IV lidocaine use in rib fracture patients (N = 12). *Missing*, centres that did not answer.

Six (50%) of the responding trauma centre directors described having an analgesic protocol for traumatic rib fracture patients (Table 2). Four (33%) of those centres uploaded a copy of their protocol. (See Appendix, Figure 1 and Figure 2 for protocol samples). The remaining two (16%) trauma centre directors who answered “yes” to having a protocol described their pain management protocol for traumatic rib fracture patients in the survey’s free-form text box. Most centres with protocols determine risk stratification using the rib fracture score and initiate multimodal analgesia, with escalation to acute pain service consultation for consideration of other analgesic modalities.

The medical directors of six trauma centres (50%) without an analgesic protocol for traumatic rib fracture patients ranked the centre’s preference for pain management strategies from a list. All respondents without a protocol reported using NSAIDs, acetaminophen, and systemic opioids in their pain management strategies for rib fractures; specifically, all respondents ranked ‘NSAIDS and acetaminophen’ as their first-choice option. The majority then ranked ‘systemic opioids’ as their second-choice option; only one respondent ranked it as their third choice (Figure 1).

Medical directors of trauma centres with a protocol were not asked to rank their preference for pain management strategies. However, consistent with the ranked preferences, all 12 (100%) surveyed centres use NSAIDs and acetaminophen as first-line management. In most centres (7/12; 58%), the trauma team is responsible for pain management in patients with traumatic rib fractures (Table 2).

### Intravenous Lidocaine Infusions

When asked whether the centre could run an IV lidocaine infusion in trauma patients, medical directors provided the following responses: ‘Yes’, 6/12 (50%); ‘Yes, but limited to certain clinical areas’, 3/12 (25%); and ‘No’, 3/12 (25%). Regarding the use of IV lidocaine for patients with traumatic rib fracture, 4/12 (33%) medical directors reported frequent use; 2/12 (16%) reported occasional use; 2/12 (16%) reported rare use; and 4/12 (33%) reported never using IV lidocaine (Figure 2). When asked how often IV lidocaine infusions are used in patients with ≥ 2 rib fractures, 3/12 (25%) reported frequent use. However, when asked how often IV lidocaine infusions were used in traumatic rib-fracture patients with flail chest, 5/12 (41%) centres responded that they frequently use IV lidocaine (Figure 2).

None of the surveyed trauma centres use IV lidocaine for first-line management. When asked under what circumstances IV lidocaine is used, medical directors provided the following responses: ‘routinely but not first line’, 5/12 (41%); and ‘infrequently’, 5/12 (41%). Two (16%) centres did not answer the question.

### Future Study

Table 3 outlines Canadian trauma centre responses to dichotomous questions surrounding pain management for traumatic rib fractures, the need for future research, and willingness to participate in a future study. Most (10/12; 83%) respondents reported agreeing that further research regarding IV lidocaine infusions for pain management is needed and are interested in being part of a multi-centre randomized controlled trial examining the effectiveness of IV lidocaine infusions in patients with traumatic rib fracture (Table 3).

## DISCUSSION

Our survey demonstrated that all responding trauma centres currently use NSAIDs and acetaminophen as first-line management for traumatic rib fractures. This is consistent with current practice patterns described in the literature.^11^ Several other trauma centre directors cited acetaminophen as the first-line therapy for traumatic rib fracture patients due to its pain-relieving and opioid-sparing effects.^25^ Furthermore, the survey identified a lack of protocols as well as variations in existing analgesic protocols. Uploaded protocols reveal the use of different risk-stratification scores and procedural steps, further suggesting the absence of an optimal protocol. This lack of an optimal protocol and variation in protocols is expected, given the challenges of current analgesic modalities and their potential to exacerbate rib fracture complications.^11^,^12^

Importantly, our data also highlight the practices of Canadian trauma centres in their administration of IV lidocaine. Most surveyed centres demonstrated the ability to run IV lidocaine infusions in trauma patients and in patients with traumatic rib fractures, underscoring the applicability and generalizability of this analgesic modality for the Canadian trauma population. In fact, 8 of 12 (66%) respondents reported using IV lidocaine infusions for rib fracture treatment. Consideration of IV lidocaine infusion can also be found in the protocols provided in the Appendix, promoting the potential relevance of lidocaine as mainstay practice.

Multiple meta-analyses have emphasized lidocaine’s effect on lowering opiate requirements and improving recovery indices following abdominal surgery.^22^,^26^,^27^ Nearly half the centres (41%) revealed frequent use of IV lidocaine in the escalation of traumatic rib fracture patients with flail chest, suggesting it may be beneficial in conditions deemed high risk for rib fracture complications, including compromised respiratory function. Previous literature suggests that an effective analgesic can reduce rib fracture-associated mortality.^28^ Altogether, if the efficacy of IV lidocaine observed in thoracic surgery patients can be demonstrated in traumatic rib fracture patients, it can safely improve their pain control and complication risks.

Half (50%) of the respondents expressed dissatisfaction with the level of analgesia currently provided, and most (83%) respondents agreed that further research is needed in Canadian trauma patients regarding the use of IV lidocaine infusions for pain management.

In 2022, a single-centre, double-blind, randomized controlled trial evaluated the analgesic efficacy of a 72- to 96-hour IV lidocaine infusion. Pain scores were improved, patient satisfaction was higher, and morphine equivalent use was lower in the lidocaine group compared to the placebo group. However, these results did not reach statistical significance.^8^ Despite this, the observed beneficial trends of the study, combined with the results of our survey-based study and respondents’ support, a multi-centre trial evaluating the analgesic efficacy and safety of IV lidocaine infusions for rib fracture pain management is now warranted.

## LIMITATIONS

The survey-based nature of this work is subject to several potential biases, including sampling bias, question-order bias, demand bias, non-response bias, response bias, and confirmation bias. The survey was completed by the medical director of each major Canadian trauma centre to represent the practice of the trauma centre and its trauma practitioners. While they were reminded to answer survey questions based on their centre’s common practice, personal biases and prior knowledge may have impacted responses regarding the centre’s standard practice. Additionally, participants were informed and given study questions that addressed the variation in practices regarding differences in trauma centre characteristics, pain management in the setting of trauma, and the role of IV lidocaine. The theme of questions, question options and increasing the role of IV lidocaine in treatment of rib fracture pain may elicit more extreme responses that support or oppose its use. Finally, the survey was also not piloted prior to distribution, potentially affecting the clarity of certain questions and resulting in misinterpretations or inaccuracies in the responses. Nonetheless, previous investigations and a randomized controlled trial support IV lidocaine as a beneficial analgesic for patients with rib fracture despite its varied use and potentially biased support.^8^,^19^,^20^

## CONCLUSION

Traumatic rib fracture-management practices across Canadian trauma centres are variable. There was majority agreement among respondents in our study that further research on IV lidocaine for pain control in traumatic rib fractures is needed. Survey respondents also expressed interest in participating in a multi-centre randomized controlled trial examining the effectiveness of IV lidocaine infusions in these patients.

## Supplementary Information





## Figures and Tables

**Table 1:** Characteristics of 12 Canadian trauma centres.

Characteristic	N (%)
Total respondents	12 (N = 12)
Annual number of severe (ISS>12) trauma admissions	
51–250	2 (16)
251–450	4 (33)
>450	6 (50)
Dedicated trauma team to assess emergency department patients?	
Yes	12 (100)
No	0
Dedicated trauma service for admitted multi-system trauma patients?	
Yes	10 (83)
No	2 (16)
Percentage of trauma patients admitted	
20–40%	1 (8)
61–80%	4 (33)
> 80%	7 (58)
Available Trauma Clinic for follow-up?	
Yes	10 (83)
No	2 (16)

Note: Severe trauma admissions include patients admitted via trauma team activation.

*ISS*, Injury Severity Score.

**Table 2:** Categorical pain management characteristics for traumatic rib fracture patients

Characteristic	N (%)
Presence of traumatic rib fracture analgesic protocol?	
Yes	6 (50)
No	6 (50)
Pain management administrator for traumatic rib fracture patients	
Trauma Team	7 (58)
Acute Pain Service	4 (33)
Admitting service	1 (8)

**Table 3:** Canadian trauma centres’ interest in future studies on use of intravenous lidocaine for rib fracture pain.

Opinion	Yes N (%)	No N (%)
Canadian-specific trauma research is important to develop, since much of the current trauma literature is derived from American trauma centres that serve very different populations with different injury patterns than we do in Canada.	10 (83)	2 (16)
Aggressive pain management is important in patients with traumatic rib fractures.	12 (100)	0
Satisfied with the level of analgesia currently provided to your trauma patients with severe thoracic trauma/multiple rib fractures.	6 (50)	6 (50)
Further research is needed in Canadian trauma patients regarding the use of IV lidocaine infusions for pain management.	10 (83)	2 (16)
In patients with traumatic rib fractures, randomizing to IV lidocaine infusion versus usual care is ethically justified.	10 (83)	2 (16)
Interest in being part of a multi-centre randomized controlled trial examining the effectiveness of IV lidocaine infusions in traumatic rib fracture patients.	10 (83)	2 (16)

*IV*, intravenous.
