# Supplementary material for: Examining Canadian Trauma Centres’ Analgesic Protocols for Rib Fractures
Source: West J Emerg Med. 2025 Sep 25;26(5):1367–73. doi: 10.5811/westjem.24945 (PMC12591657; doi:10.5811/westjem.24945)
Supplement: Supplementary file 1 [file wjem-26-1367-s001.pdf]

The Ottawa Hospital Pathway for Early Intervention in Rib Fracture

STEP 1: Calculate Rib # Score

Rib Fracture Score = Breaks x Sides + Age Factor *Adapted from Easter (2001)*		
**Breaks**	**Sides**	**Age Factor**
Total # of Fractures to the ribs *(Not the # of ribs fractured)	Unilateral = 1<br>Bilateral = 2	<50 years = 0<br>51-60 years = 1<br>61-70 years = 2<br>71-80 years = 3<br>>80 years = 4

Rib # Score ≤6 & No supplemental O2 → Optimize multi-modal PO analgesia

- Acetaminophen 1000mg q6h
- +/- NSAID (Celebrex 200mg BID)
- PO/SC opioid
  - +/- Tramadol 50mg q8h
  - Hydromorphone 1-2mg PO q4h PRN
- +/- Gabapentinoid
  - Pregabalin 25-50mg q8h

Rib # score ≥7 OR ≤6 + Supplemental O2 → Consult APS + Multi-modal analgesia +/- Regional +/- PCA

- +/- Ketamine infusion
- +/- Lidocaine infusion

  Consider when regional not possible
- Serratus Anterior Plane Catheter[Ψ] OR Thoracic epidural* (when expertise available)
  - ➢ PVB/catheter – alternative (provider discretion)
- +/- IV PCA

Rib # Score >10

- Consider CPAP/BiPAP

[Ψ]**SAP Catheter**: Not well studied, but clinical benefit seen. Consider for unilateral #'s +/- position issues +/- anticoagulated patients, when expertise permits. *(Run catheter at 8ml/h + 8ml bolus q30 mins OR nurse administered bolus 8ml q3h)*

***Epidural**: Multiple studies show improved pulmonary function, pain control, and better clinical outcomes vs systemic opioids
