# Supplementary material for: Examining Canadian Trauma Centres’ Analgesic Protocols for Rib Fractures
Source: West J Emerg Med. 2025 Sep 25;26(5):1367–73. doi: 10.5811/westjem.24945 (PMC12591657; doi:10.5811/westjem.24945)
Supplement: Supplementary file 2 [file wjem-26-1367-s002.pdf]

Pain Control & Respiratory Management for Chest Trauma Algorithm
Initiate Upon Admission

Assess Pain and Risk Stratification

Rib Fracture Score (RFS)
(Breaks x Sides) + Age Factor = RFS

- Breaks = # of fractures
- Sides: Unilateral = 1 Bilateral = 2
- Age Factor
  - 0 if < 50
  - 1 if 51-60
  - 2 if 61-70
  - 3 if 71-80
  - 4 if > 80 years

*if ≥65 years & ≥3 rib #s pt is considered High Risk

Score 3-6

Step 1
- Regular acetaminophen
- +/- NSAIDs
- +/- oral hydromorphone prn
- Hydromorphone (IV/SC)
- Antiemetics

Is pain controlled (< 6/10) within 4 hours?

YES → Continued established analgesia regime → Observe and Reassess prn

NO → Step 2

Score >6 or High Risk

Step 2
- Regular acetaminophen
- +/- NSAIDs
- +/- oral hydromorphone prn
- Hydromorphone (IV/SC)
- Antiemetics

AND

- **Referral to APS for consideration of:**
  - IV Opioid via PCA
  - Gabapentinoid
  - Antiemetics
  - Regional or Neuraxial technique

Assess the Need for Respiratory Support

PIC Score (Modified)

**Pain Score** (Scale 0-10)	**Inspiration (Vital Capacity)** Goal is 20 ml/kg	**Cough Strength**
1 – Controlled (0 - 4)	1 – Above Goal Volume	1 – Strong
2 – Moderate (5 - 7)	2 – Goal Volume	2 – Weak
3 – Severe (8 - 10)	3 – Below Goal Volume<br>4 – Unable to Perform	3 – Absent

PIC Score 3-5
Reassess Q6h for 48 hrs

PIC Score ≥9
- Notify Physician
- Consult ICU / HAU

PIC Score 6, 7, 8
- Consult ICU/HAU
- Begin BiPAP **
- NPO
- Reassess PIC Score Q6h and prn for 48hrs

****BiPAP contraindicated if**
- PIC Score ≤ 4
- GCS <14
- Vomiting
- Facial injuries
- Suspected or confirmed COVID-19 positive (refer to COVID risk stratification algorithm)

Anytime there is 6 hours of poor pain mgmt Initiate: Physician to Physician consult & Reassessment by APS
